# A Systematic Review and Meta-Analysis of the Performance of Two Point of Care Typhoid Fever Tests, Tubex TF and Typhidot, in Endemic Countries

**DOI:** 10.1371/journal.pone.0081263

**Published:** 2013-12-16

**Authors:** Kamala Thriemer, Benedikt Ley, Joris Menten, Jan Jacobs, Jef van den Ende

**Affiliations:** Department of Clinical Sciences, Institute of Tropical Medicine, Antwerp, Belgium; Cincinnati Children's Hospital, United States of America

## Abstract

**Background:**

In the absence of well-equipped laboratory infrastructure in many developing countries the accurate diagnosis of typhoid fever is challenging. Rapid diagnostic tests (RDT) with good performance indicators would be helpful to improve clinical management of suspected cases. We performed a systematic literature review and meta- analysis to determine the performance of TUBEX TF and Typhidot for the diagnosis of typhoid fever using PRISMA guidelines.

**Methods:**

Titles and abstracts were reviewed for relevance. Articles were screened for language, reference method and completeness. Studies were categorized according to control groups used. Meta-analysis was performed only for categories where enough data was available to combine sensitivity and specificity estimates. Sub-analysis was performed for the Typhidot test to determine the influence of indeterminate results on test performance.

**Results:**

A total of seven studies per test were included. The sensitivity of TUBEX TF ranged between 56% and 95%, Specificity between 72% and 95%. Meta-analysis showed an average sensitivity of 69% (95%CI: 45–85) and an average specificity of 88% (CI95%:83–91). A formal meta-analysis for Typhidot was not possible due to limited data available. Across the extracted studies, sensitivity and specificity estimates ranged from 56% to 84% and 31% to 97% respectively.

**Conclusion:**

The observed performance does not support the use of either rapid diagnostic test exclusively as the basis for diagnosis and treatment. There is a need to develop an RDT for typhoid fever that has a performance level comparable to malaria RDTs.

## Introduction

*Salmonella enterica* serovar Typhi (*Salmonella* Typhi), the causative agent of typhoid fever, has been estimated to have caused more than 21.000.000 episodes of typhoid fever at a 1% mortality rate in the year 2000 [1]. The major disease burden lies in developing countries.

Due to the lack of reliable diagnostic tools the estimated incidence rate may be an underestimate for the African continent, as more recent data indicate [2], [3]. Since typhoid fever has a non-specific clinical picture [4], [5], accurate diagnosis remains a challenge in resource poor settings [6]. Blood culture is the current reference method for diagnosis, however results are only available after >48 hours, the procedure is expensive and requires extensive laboratory equipment and technical expertise. Sensitivity is estimated to be between 40% and 70% [7], [8], [9], [10], [11], [12]. Culture from bone marrow is known to be more sensitive [8], [9], [10], however the invasive character renders the procedure inappropriate for large scale application. Rapid diagnostic tests (RDTs) with good performance indicators at a low price are therefore desirable to provide a reliable diagnosis.

Typhidot (Malaysian Biodiagnostic Research, Malaysia) and Tubex TF (IDL, Sweden) are among the most widely used RDTs within the more recently developed diagnostic devices for typhoid fever. There are a number of other test available such as the Typhidot-M (Malaysian Biodiagnostic Research, Malaysia), the Multi-Test Dip-S-Ticks (Panbio INDX, US), SD Bioline (Standard Diagnostics, Korea) and Mega Salmonella (Mega Diagnostics, US) however little data on their performance is available [13], [14], [15], [16].

Tubex TF is based on an inhibition reaction between patient antibodies (IgM) and monoclonal antibodies included in the test that bind to a *Salmonella* Typhi specific O9 lipopolysaccharide. A macroscopically visible de-colorization of patient serum in test reagent solution through magnetic particle separation indicates a positive result. In contrast the Typhidot is based on a qualitative dot-blot enzyme-linked immunosorbent assay that separately detects the presence of IgM and IgG in patient sera against a *Salmonella* Typhi specific 50 kD outer membrane protein.

Several studies have assessed the performance of either test for the diagnosis of symptomatic patients, but no formal meta-analysis of the available data has been performed to date.

We therefore aimed to analyze the diagnostic performance of Tubex TF (IDL, Sweden) and Typhidot (Malaysian Biodiagnostic Research, Malaysia) for the diagnosis of typhoid fever in patients in typhoid endemic regions.

## Methods

### General

We performed a review and meta- analyses using the PRISMA guidelines [17] for systematic reviews and meta-analyses (Checklist S1).

### Search method and inclusion criteria

We performed a literature search in the MEDLINE database through PubMed using “Tubex” and “Typhidot” as search terms. Searches were restricted to publications from 1998 to date to cover the time since introduction of either test to the market. In addition we conducted supplementary searches in the references of the retrieved articles. Titles and abstracts were reviewed for relevance.

Only articles evaluating the performance of one of the two or both test were included. Articles were excluded based on title, abstract, language other than English, lack of automated blood culture as reference method assuming that automated blood culture has a better yield in patients with previous antimicrobial treatment and to assure standardization across the different studies [18]. Articles were further excluded because presented data was insufficient and authors did not reply to our queries. Whenever automated and manual blood culture had been used as reference method, only results of the automated blood culture were included. Corresponding authors were contacted via email for additional information whenever necessary. Information provided by the authors was anonymized. If no answer was provided within eight weeks of the first email and two additional follow up emails (sent without an error report) the respective studies were excluded.

### Data retrieval and definitions

The number of true positives (TF), true negatives (TN), false positives (FP) and false negatives (FN) were retrieved from each article by two investigators independently and entered into an excel datasheet. Discordant findings were assessed in a joint approach and authors asked for verification when in doubt. We obtained sensitivity, specificity and accuracy estimates of each included study considering blood culture as the reference method. Sensitivity was defined as the number of true positive results per true positive and false negative results 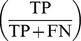. Specificity was defined as the number of true negative results per true negative and false positive results 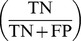. Accuracy was defined as the number of true results divided by the total sample size 
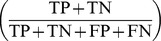
.

To address poor sensitivity of blood culture [7], [8], [9], [10], [11], [12] we repeated the analysis applying different control groups whenever possible. Control groups to determine true negatives were defined as follows: category 1 – samples with known etiology other than *Salmonella* Typhi; category 2 – samples with unknown etiology (blood culture negative); category 3 – categories 1 and 2 combined.

Results for IgM and IgG for the Typhidot where assessed separately. Whenever articles evaluating the Typhidot did not present results for IgG and IgM separately, authors were contacted and asked to provide respective data. Based on these data the following outcomes were defined: presence of IgM alone = positive (diseased); presence of both IgG and IgM = positive; absence of both IgG and IgM = negative; presence of IgG alone = indeterminate. If information regarding the number of indeterminate results among cases and controls was not provided in the article the respective numbers were retrieved from the authors.

### Risk of bias

The QUADAS checklist [19] has been completed for all included studies (Table S1). Given the limited number of studies included, we did not perform a sensitivity analyses excluding lower quality studies. However sensitivity analyses for the most likely source of bias, the handling of indeterminate results has been performed as described below.

### Tubex TF

For Tubex TF we plotted estimates of the sensitivity and specificity in forest plots as well as receiver-operating characteristic (ROC) space using RevMan 5 [20] for each category. Meta-analysis was performed only for categories where enough data was available to produce average sensitivity and specificity estimates. Estimates were calculated using logistic regression separately for sensitivity and specificity correcting for heterogenity among studies using robust standard errors (generalized estimating equations), an approach similar to random effects meta-analysis [21].

### Typhidot

For Typhidot no formal meta-analysis was performed, firstly due to the low number of studies included in each control group and secondly because information on the inclusion or exclusion of indeterminate results could not be retrieved for all studies. For studies where information on the number of indeterminate results was available, sensitivity, specificity and accuracy estimates were calculated using three different approaches:

Firstly we excluded the indeterminate results completely from the analyses, Secondly we defined the indeterminate results as negative results (TN and FN respectively):
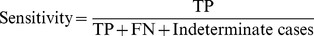


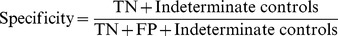
Thirdly we added indeterminate results only to the denominator, resulting in a new formula for specificity only but not for sensitivity when compared to the second approach:
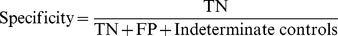
For studies where information on the number of indeterminate results was not available the results are presented as given by the respective authors.

95% Confidence intervals were calculated according to Wilsons score method and the difference between accuracy estimates was calculated using chi2 test considering p<0.05 as significant.

## Results

**Figure 1:**
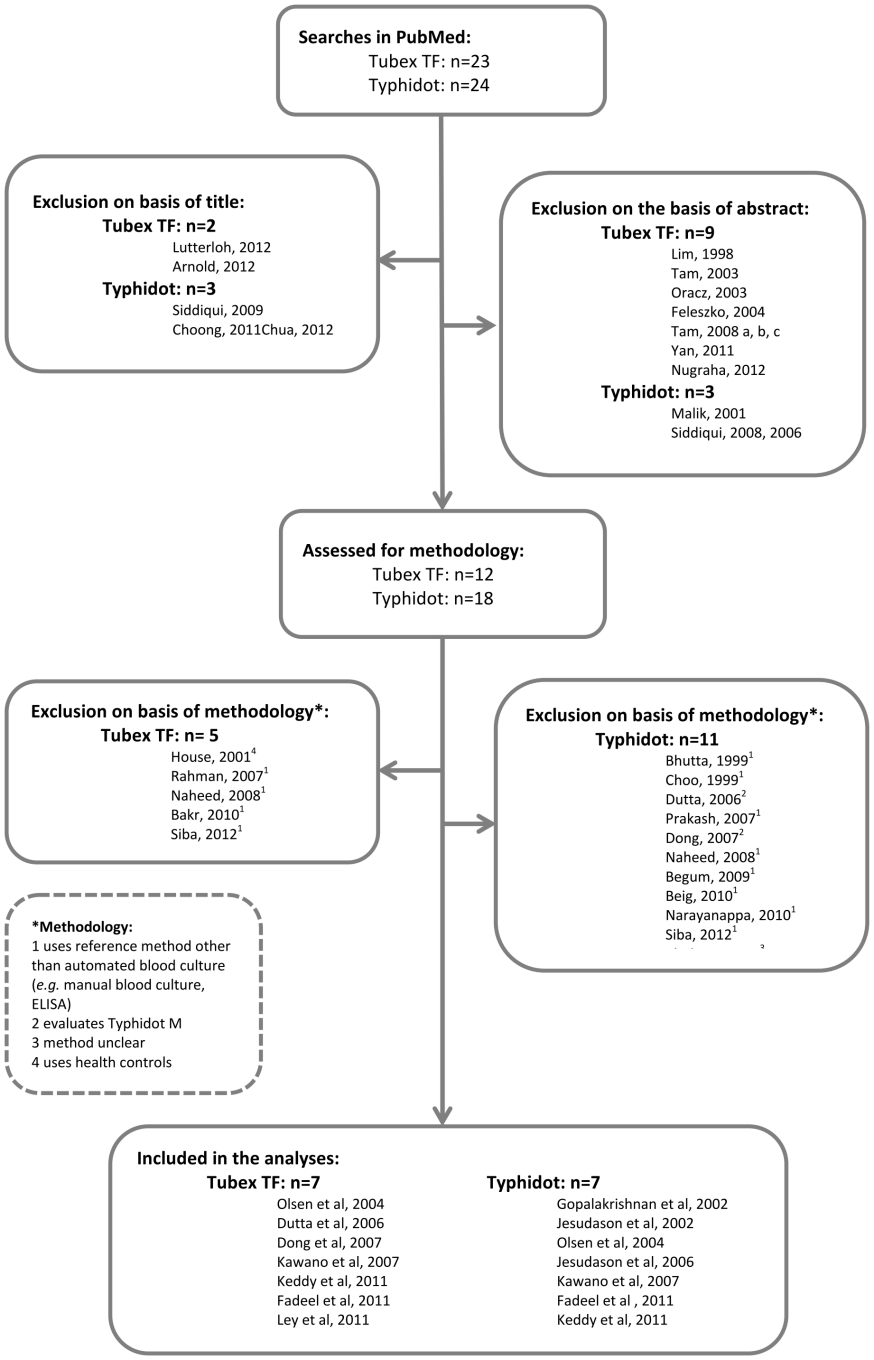
Selection of studies included in the analyses.

The search word “Tubex” retrieved 23 hits, “Typhidot” retrieved 24 hits. Based on the title we excluded two articles (8.7%) for the evaluation of the Tubex TF test and three (12.5%) for the Typhidot. For Tubex TF nine articles (39.1%) and for Typhidot three (12.5%) were excluded based on the abstract. Respectively five (17.4%) [22], [23], [24], [25], [26] and 11 (45.8%) [24], [26], [27], [28], [29], [30], [31], [32], [33], [34] studies were excluded as the underlying methods did not fit our predefined criteria (Figure 1). The QUADAS checklist revealed that risk of bias could generally be considered low for all studies (Table S1).

### Tubex TF

A total of seven (30.4%) studies evaluating Tubex TF with different control groups were included in the analyses. One of the studies used two different control groups as comparison and was therefore included in two different categories [35] with the respective results. A total of five studies using febrile controls with known etiology [13], [29], [31], [35], [36] were therefore included in category 1, two studies using controls with unknown febrile diseases [15], [35] were included in category 2 and one study that used controls with known and unknown etiology [37] was included in category 3. Characteristics of the studies are summarized in Table 1.

**Table 1:** Characteristics of included studies.

Study	Test evaluated (Typhidot/Tubex)	Control group used*	Additional data received from the authors (Yes/No)	Country
Dong et al, 2008 [31]	Tubex	1	No	Southern China
Dutta et al, 2006 [29]	Tubex	1	No	Calcutta, India
Fadeel et al, 2011 [35]	Typhidot/Tubex	1; 2	Yes	Egypt
Gopolaskrishnan et al, 2002 [14]	Typhidot	1; 2	Yes	Kuala Lumpur, Malaysia
Jesudason et al, 2002 [38]	Typhidot	2	Yes	Vellore, India
Jesudason et al, 2006 [39]	Typhidot	3	Yes	Vellore, India
Kawano et al, 2007 [15]	Typhidot/Tubex	2	Yes	Philippines
Keddy et al, 2011 [37]	Typhidot/Tubex	3	Yes	South Africa/Tanzania
Ley et al, 2011 [36]	Tubex	1	No	Tanzania
Olsen et al, 2004 [13]	Typhidot/Tubex	1	Yes	Ho Chi Min city, Vietnam

*controls groups: 1 – samples with known etiology other than *Salmonella* Typhi; 2 – samples with unknown etiology (blood culture negative); 3 – a combination of 1 and 2.

**Figure 2:**
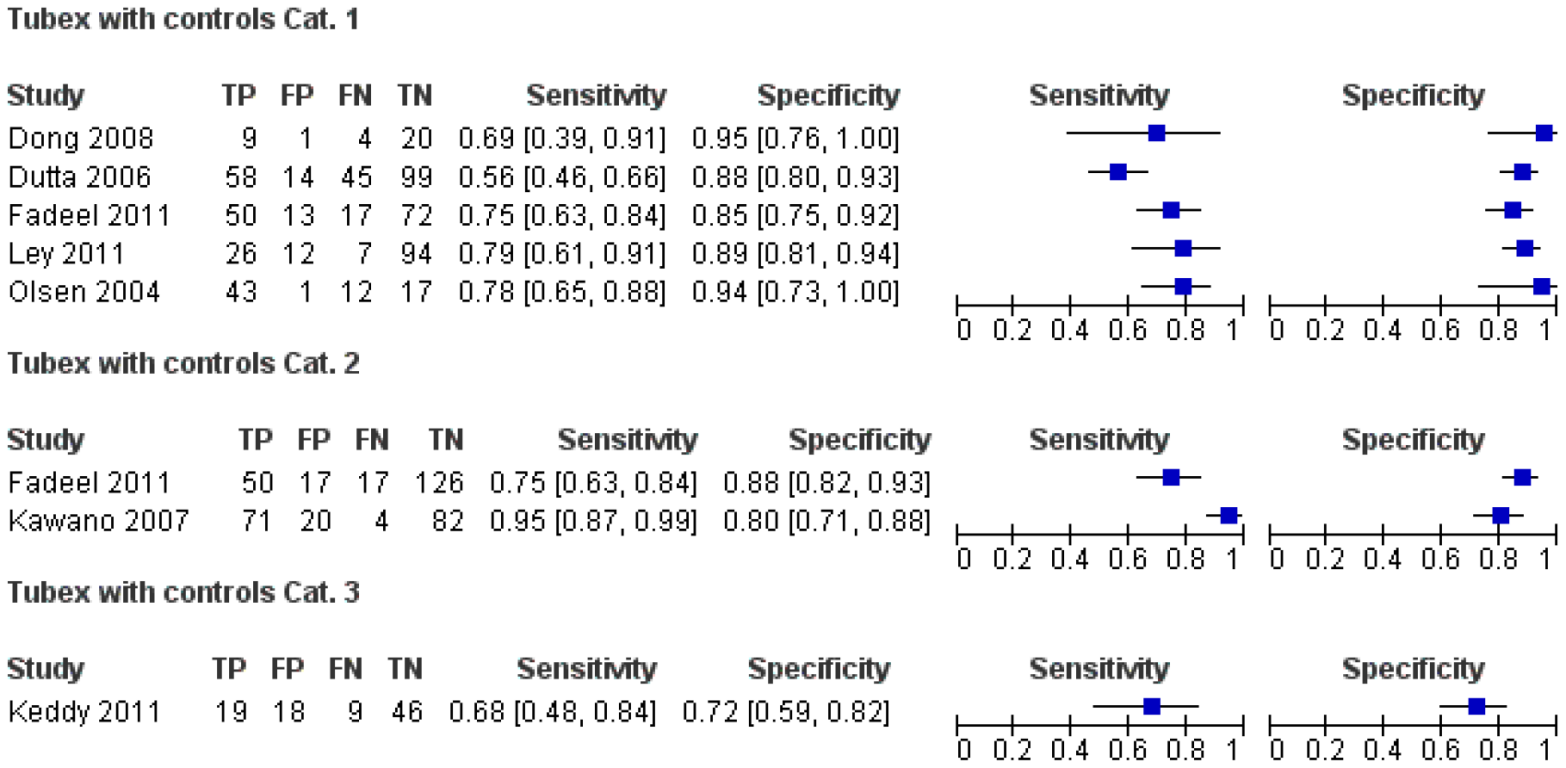
Forest Plot for Tubex TF with different control groups.

Sensitivity of Tubex TF in the studies included in category 1 varied between 56% [29] and 79% [36], specificity between 85% [35] and 95% [31]. Sensitivity and specificity for studies included in category 2 were 75% [35] and 95% [15] and 88% [35] and 80% [15] respectively and the study in category 3 showed sensitivity of 68% and specificity of 72% [37] (Figure 2).

**Figure 3:**
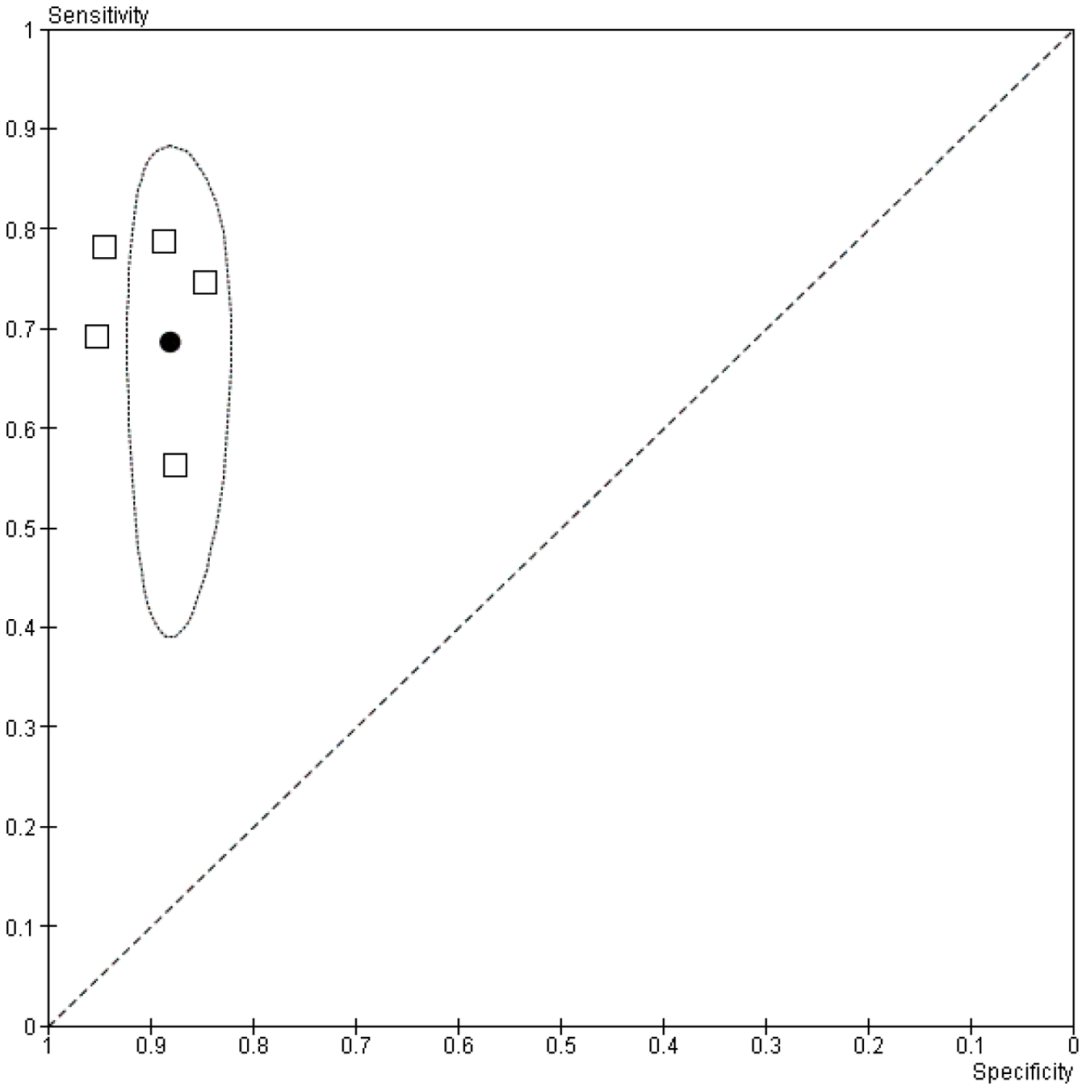
Sensitivity and specificity estimates for Tubex TF (open squares) with control group category 1 together with average sensitivity and specificity estimate (filled circle) and 95% confidence region.

Meta-analysis of the data in category 1 showed an average sensitivity of 69% (95%CI: 45–85) and a specificity of 88% (CI95%:83–91)(Figure 3). No meta-analysis was performed for the other categories due to the low number of studies included.

### Typhidot

For the evaluation of the Typhidot a total of seven (29.2%) studies were included in the analyses. Two studies used two different control groups and were therefore included in two categories with the respective results [14], [35]. Therefore a total of three studies could be included in category 1 [13], [14], [35], four studies in category 2 [14], [15], [35], [38] and two studies in category 3 [37], [39]. Additional characteristics of the included studies are shown in Table 2.

**Table 2:** Overview Typhidot studies.

	Comment
**Typhidot with control group 1**	
Fadeel, 2011	-Results are originally presented for IgG and IgM separately. Additional data provided by the author.
Gopalakrishnan, 2002	-No information on the number of indeterminate results/not specified how they were counted.
Olsen, 2004	-Results are originally presented for 2 hospitals combined. We only included data using automated blood culture as reference method as provided by the author.
**Typhidot with control group 2**	
Fadeel, 2011	-Results are originally presented for IgG and IgM separately. Additional data provided by the author.
Gopalakrishnan, 2002	-No information on the number of indeterminate results/not specified how they were counted.
Jesudason, 2002	-No information on the number of indeterminate results/not specified how they were counted.
Kawano, 2007	-Results are originally presented for IgG and IgM separately. Additional data provided by the author.
**Typhidot with control group 3**	
Jesudason, 2006	-No information on the number of indeterminate results/not specified how they were counted.
Keddy, 2011	-Additional data provided by author.

The number of indeterminate results (presence of IgG alone) obtained when using Typhidot showed a great variation among studies. Kawano *et al.* [15] reported 55 indeterminate results (out of 366 total results) both among cases and controls respectively. Fadeel *et al.* [35] reported five indeterminate samples among cases and one among controls (out of a total of 140 and 210 results depending on the control group), Olsen *et al.* [13] reported six indeterminate results, three among cases and three among controls (out of a total of 77 results), Keddy *et al.* [37] reported no indeterminate results (out of 80 results).

Depending on how indeterminate results are classified sensitivity and specificity can vary. Highest numbers of indeterminate results for the Typhidot were reported by Kawano *et al* [15] with a total of 30% of all results being indeterminate. Accordingly sensitivity of the test was 82% when indeterminate results were excluded, 56% when the respective results were considered negative and 56% when indeterminate results were included in the denominator. Accordingly specificity was 44%, 60% and 31% respectively (p<0.05 for accuracy).

For studies with smaller numbers of indeterminate results no significant differences in accuracy were found and sensitivity varied between 63% and 84%, specificity between 74% and 97% depending on control group and definition of indeterminate results (Table 3). Results from studies were no information on indeterminate results were available are listed in Table 4.

**Table 3:** Sensitivity (Sens.), Specificity (Spec.) and Accuracy (Acc.) of Typhidot depending on definition of indeterminate results (only studies included where information on indeterminate results are available).

							Indeterminate results excluded			Indeterminate results counted as negatives			Indeterminate results only included in the denominator			
	TP	FP	FN	TN	Indet. among cases	Indet. among controls	Sens. (95%CI)	Spec. (95%CI)	Acc. (95%CI)	Sens. (95%CI)	Spec. (95%CI)	Acc. (95%CI)	Sens. (95%CI)	Spec. (95%CI)	Acc. (95%CI)	P (Acc.)
**Typhidot with control group category 1** *																
Fadeel, 2011	42	4	20	68	5	1	0.68 (0.55–0.79)	0.94 (0.86–0.99)	0.82 (0.75–0.88)	0.63 (0.51–0.73)	0.95 (0.87–0.98)	0.79 (0.72–0.86)	0.63 (0.51–0.73)	0.93 (0.85–0.97)	0.79 (0.71–0.85)	0.968
Olsen, 2004	46	2	9	14	3	3	0.84 (0.71–0.92)	0.88 (0.62–0.99)	0.85 (0.74–0.92)	0.79 (0.67–0.89)	0.89 (0.67–0.99)	0.82 (0.71–0.90)	0.79 (0.67–0.89)	0.74 (0.51–0.88)	0.78 (0.67–0.87)	0.946
**Typhidot with control group category 2** *																
Fadeel, 2011	42	5	20	137	5	1	0.68 (0.55–0.79)	0.96 (0.92–0.99)	0.88 (0.82–0.92)	0.63 (0.50–0.74)	0.97 (0.92–0.99)	0.86 (0.80–0.90)	0.63 (0.50–0.74)	0.96 (0.91–0.98)	0.85 (0.80–0.90)	0.978
Kawano, 2007	97	78	21	60	55	55	0.82 (0.74–0.89)	0.44 (0.35–0.52)	0.61 (0.55–0.67)	0.56 (0.48–0.64)	0.60 (0.52–0.67)	0.58 (0.58–0.63)	0.56 (0.48–0.64)	0.31 (0.25–0.38)	0.43 (0.38–0.48)	**0.018**
**Typhidot with control group category 3** *																
Keddy, 2011	19	13	8	40	0	0	0.70 (0.50–0.86)	0.75 (0.62–0.83)	0.74 (0.63–0.83)	0.70 (0.50–0.86)	0.75 (0.62–0.86)	0.74 (0.63–0.83)	0.70 (0.50–0.86)	0.75 (0.62–0.85)	0.74 (0.63–0.83)	N/A

*category 1 – samples with known etiology other than *Salmonella* Typhi; category 2 – samples with unknown etiology (blood culture negative); category 3 – a combination of category 1 and 2.

**Table 4:** Sensitivity and Specificity of Typhidot in studies where no information in indeterminate results was available.

	TP	FP	FN	TN	Sensitivity (95%CI)	Specificity (95%CI)
**Typhidot with control group category 1** *						
Gopalakrishnan, 2002	41	12	9	32	0.82 (0.69–0.91)	0.73 (0.57–0.85)
**Typhidot with control group category 2** *						
Gopalakrishnan, 2002	41	18	9	32	0.82 (0.69–0.91)	0.64 (0.49–0.77)
Jesudason, 2002	30	6	0	24	1.00 (0.88–1.00)	0.80 (0.61–0.92)
**Typhidot with control group category 3** *						
Jesudason, 2006	36	6	3	506	0.92 (0.79–0.98)	0.99 (0.97–1.00)

*category 1 – samples with known etiology other than *S*.Typhi; category 2 – samples with unknown etiology (blood culture negative); category 3 – a combination of category 1 and 2.

## Discussion

Our meta-analysis for Tubex TF showed an average sensitivity of 69% and a specificity of 88%. Even though no meta-analysis was performed for the Typhidot, sensitivity and specificity varied between 46% and 79% and 31% and 96% respectively when including indeterminate results in the denominator only and across all three control groups. The number of indeterminate results varied between 0% [37] and 30% [15] of the entire study population. However we found that apart from the study conducted by Kawano *et al.* [15] the number of indeterminate results was low and did not significantly affect test accuracy (p>0.05) (Table 3). This study only considered sensitivity, specificity, and accuracy for analysis but not predictive values. Predictive values are much heavier affected by prevalence of disease within the study population than sensitivity and specificity, making it difficult to compare predictive values of different studies.

Malaria and typhoid fever may be considered among the most mportant non-viral infectious diseases in developing countries. For malaria a plethora of RDTs is available and current WHO recommendations for the use of those RDTs as an exclusive method of diagnosis postulate a specificity >90% in order to be used on a wider scale [40]. While the average performance of the Tubex TF does not qualify according to these criteria, few individual studies for Tubex TF [29], [31] and Typhidot [35], [39] report performance above the given threshold.

Since typhoid fever is a potentially fatal disease, easily treatable with affordable antibiotics, its treatment threshold is very low. Moreover no clinical signs with sufficient predictive value are available, and consequently in most situations the disease is treated presumptively. In order for a typhoid RDT to be superior to presumptive treatment, a respective test would require a high sensitivity, in order not to miss possibly fatal cases. On the other hand, even a moderate specificity will allow avoiding the many false positives inherent to the presumptive strategy, leading to unnecessary antibiotics overuse, resulting in resistance on a population scale. The question remains, if RDT's based on antibodies are sufficiently sensitive for an early presentation. Malaria tests are based on antigen detection, an approach that yields positive results earlier after infection, as the result is not delayed by a host immunological response.

Parry *et al.* [41] suggest testing paired samples to improve performance of the RDTs. Assuming that false positive results occur on an independent basis, this will increase specificity. Likewise, if samples are taken at a timely interval this is likely to improve sensitivity due to higher antibody titers within the course of the disease. The latter approach might be useful for epidemiological purposes but its value in a clinical setting is limited.

The major limitation of the presented data is the small number of study results available. While sufficient publications were retrieved to calculate performance indicators for the Tubex TF test, this was not possible for Typhidot. Different methods in defining and including controls have made it difficult to standardize earlier collected data and have further reduced the number of data that we could compare directly.

The unknown sensitivity of blood culture is likely to have affected the analyzed results. We excluded all studies where manual blood culture was used as a reference method, assuming that automated blood culture has a higher yield in patients with previous antibiotic treatment and to assure some standardization of the workflow across the different studies included [18]. However also automated blood culture results are dependent on skills and knowledge of the performing laboratory staff as well as the condition of local laboratory equipment and consumables. Moreover choosing the most appropriate control group for an RDT evaluation remains a challenge when blood culture is the reference method. Including blood culture negative patients in the control group bears the risk of including undetected *Salmonella* Typhi cases due to poor sensitivity of blood culture among the controls affecting the specificity of the evaluated test. On the other hand including only febrile cases with a confirmed laboratory diagnosis other than typhoid fever results in an unrealistic control group.

Additional limitations in the longitudinal test evaluation are inter-batch variation as well as minor test modification by the manufactures that are not leading to changes in the brand name and not made public [42], [43]. Indeed the Tubex TF test had been modified within the evaluated time period without changes of the product name (IDL personal communication). The study from Olsen *et al* [13] had evaluated the former version of the test (IDL personal communication), however when repeating the analysis and excluding the respective publication, we found similar results for average sensitivity and specificity (data not shown).

In the light of poor sensitivity of current blood culture procedures at high costs, requiring considerable expertise and long time to diagnosis, the demand for a reliable RDT in clinical settings remains high. Apart from good performance indicators, a respective test would require good operational characteristics as well as moderate pricing comparable to currently used malaria RDTs. In addition a diagnostic device to detect *Salmonella* carriers would be a powerful tool to estimate true disease burden and potential of transmission [41].

## Conclusion

The performance of Typhidot and TUBEX TF does not support the use of either rapid diagnostic test exclusively as a basis for diagnosis and treatment. Although more time consuming and related to higher expenses and logistics, blood culture and molecular biologic techniques remain the reference method of choice, despite its limitations. There is a need to develop an RDT for typhoid fever that has a performance level comparable to malaria RDTs.

## Supporting Information

Checklist S1 **PRISMA checklist.**
(DOC)

Table S1 **QUADAS checklist.**
(XLSX)

## References

[1] Crump JA, Luby SP, Mintz ED (2004) The global burden of typhoid fever. Bulletin of the World Health Organization 82: 346–353. 15298225 PMC2622843

[2] Thriemer K, Ley B, Ame S, von Seidlein L, Pak GD, et al (2012) The burden of invasive bacterial infections in Pemba, Zanzibar. PloS one 7: e30350. 22363426 10.1371/journal.pone.0030350 PMC3281825

[3] Breiman RF, Cosmas L, Njuguna H, Audi A, Olack B, et al (2012) Population-based incidence of typhoid fever in an urban informal settlement and a rural area in Kenya: implications for typhoid vaccine use in Africa. PloS one 7: e29119. 22276105 10.1371/journal.pone.0029119 PMC3261857

[4] Mtove G, Amos B, von Seidlein L, Hendriksen I, Mwambuli A, et al (2010) Invasive salmonellosis among children admitted to a rural Tanzanian hospital and a comparison with previous studies. PloS one 5: e9244. 20168998 10.1371/journal.pone.0009244 PMC2821934

[5] Thriemer K, Ley BB, Ame SS, Deen JL, Pak GD, et al (2012) Clinical and epidemiological features of typhoid fever in Pemba, Zanzibar: assessment of the performance of the WHO case definitions. PloS one 7: e51823. 23284780 10.1371/journal.pone.0051823 PMC3527440

[6] Crump JA (2012) Typhoid Fever and the challenge of nonmalaria febrile illness in sub-saharan Africa. Clinical infectious diseases : an official publication of the Infectious Diseases Society of America 54: 1107–1109. 22357701 10.1093/cid/cis024

[7] Bhutta ZA (2006) Current concepts in the diagnosis and treatment of typhoid fever. BMJ 333: 78–82. 16825230 10.1136/bmj.333.7558.78 PMC1489205

[8] Farooqui BJ, Khurshid M, Ashfaq MK, Khan MA (1991) Comparative yield of Salmonella typhi from blood and bone marrow cultures in patients with fever of unknown origin. Journal of clinical pathology 44: 258–259. 2013632 10.1136/jcp.44.3.258 PMC496954

[9] Gilman RH, Terminel M, Levine MM, Hernandez-Mendoza P, Hornick RB (1975) Relative efficacy of blood, urine, rectal swab, bone-marrow, and rose-spot cultures for recovery of Salmonella typhi in typhoid fever. Lancet 1: 1211–1213. 48834 10.1016/s0140-6736(75)92194-7

[10] Wain J, Pham VB, Ha V, Nguyen NM, To SD, et al (2001) Quantitation of bacteria in bone marrow from patients with typhoid fever: relationship between counts and clinical features. Journal of clinical microbiology 39: 1571–1576. 11283089 10.1128/JCM.39.4.1571-1576.2001 PMC87972

[11] Parry CM, Hien TT, Dougan G, White NJ, Farrar JJ (2002) Typhoid fever. The New England journal of medicine 347: 1770–1782. 12456854 10.1056/NEJMra020201

[12] Willke A, Ergonul O, Bayar B (2002) Widal test in diagnosis of typhoid fever in Turkey. Clinical and diagnostic laboratory immunology 9: 938–941. 12093703 10.1128/CDLI.9.4.938-941.2002 PMC120044

[13] Olsen SJ, Pruckler J, Bibb W, Nguyen TM, Tran MT, et al (2004) Evaluation of rapid diagnostic tests for typhoid fever. Journal of clinical microbiology 42: 1885–1889. 15131144 10.1128/JCM.42.5.1885-1889.2004 PMC404619

[14] Gopalakrishnan V, Sekhar WY, Soo EH, Vinsent RA, Devi S (2002) Typhoid fever in Kuala Lumpur and a comparative evaluation of two commercial diagnostic kits for the detection of antibodies to Salmonella typhi. Singapore medical journal 43: 354–358. 12437043

[15] Kawano RL, Leano SA, Agdamag DM (2007) Comparison of serological test kits for diagnosis of typhoid fever in the Philippines. Journal of clinical microbiology 45: 246–247. 17065261 10.1128/JCM.01403-06 PMC1828988

[16] Anusha R, Ganesh R, Lalitha J (2011) Comparison of a rapid commercial test, Enterocheck WB((R)), with automated blood culture for diagnosis of typhoid fever. Annals of tropical paediatrics 31: 231–234. 21781418 10.1179/1465328111Y.0000000030

[17] Moher D, Liberati A, Tetzlaff J, Altman DG (2009) Preferred reporting items for systematic reviews and meta-analyses: the PRISMA statement. PLoS medicine 6: e1000097. 19621072 10.1371/journal.pmed.1000097 PMC2707599

[18] Ziegler R, Johnscher I, Martus P, Lenhardt D, Just HM (1998) Controlled clinical laboratory comparison of two supplemented aerobic and anaerobic media used in automated blood culture systems to detect bloodstream infections. Journal of clinical microbiology 36: 657–661. 9508291 10.1128/jcm.36.3.657-661.1998 PMC104604

[19] Whiting PF, Rutjes AW, Westwood ME, Mallett S, Deeks JJ, et al (2011) QUADAS-2: a revised tool for the quality assessment of diagnostic accuracy studies. Annals of internal medicine 155: 529–536. 22007046 10.7326/0003-4819-155-8-201110180-00009

[20] RMRC (2011) Version 5.1. Copenhagen: The Nordic Cochrance Centre, The Cochrance Collaboration.

[21] Egger M DSG, Altman DG, Deeks JJ, Altman DG, Bradburn MJ (2001) Statistical methods for examining and combining results from several studies in meta-analysis. In: Egger M DSG, Altman DG editor. Systematic reviews in health care. London: BMJ Books. pp. 285–311.

[22] House D, Wain J, Ho VA, Diep TS, Chinh NT, et al (2001) Serology of typhoid fever in an area of endemicity and its relevance to diagnosis. Journal of clinical microbiology 39: 1002–1007. 11230418 10.1128/JCM.39.3.1002-1007.2001 PMC87864

[23] Rahman M, Siddique AK, Tam FC, Sharmin S, Rashid H, et al (2007) Rapid detection of early typhoid fever in endemic community children by the TUBEX O9-antibody test. Diagnostic microbiology and infectious disease 58: 275–281. 17350203 10.1016/j.diagmicrobio.2007.01.010

[24] Naheed A, Ram PK, Brooks WA, Mintz ED, Hossain MA, et al (2008) Clinical value of Tubex and Typhidot rapid diagnostic tests for typhoid fever in an urban community clinic in Bangladesh. Diagnostic microbiology and infectious disease 61: 381–386. 18501549 10.1016/j.diagmicrobio.2008.03.018

[25] Bakr WM, El Attar LA, Ashour MS, El Tokhy AM (2010) TUBEX Test Versus Widal Test In The Diagnosis Of Typhoid Fever In Kafr El -Shekh, Egypt. The Journal of the Egyptian Public Health Association 85: 285–296. 22054103

[26] Siba V, Horwood PF, Vanuga K, Wapling J, Sehuko R, et al (2012) Evaluation of serological diagnostic tests for typhoid fever in Papua New Guinea using a composite reference standard. Clinical and vaccine immunology : CVI 19: 1833–1837. 22993409 10.1128/CVI.00380-12 PMC3491554

[27] Bhutta ZA, Mansurali N (1999) Rapid serologic diagnosis of pediatric typhoid fever in an endemic area: a prospective comparative evaluation of two dot-enzyme immunoassays and the Widal test. The American journal of tropical medicine and hygiene 61: 654–657. 10548305 10.4269/ajtmh.1999.61.654

[28] Choo KE, Davis TM, Ismail A, Tuan Ibrahim TA, Ghazali WN (1999) Rapid and reliable serological diagnosis of enteric fever: comparative sensitivity and specificity of Typhidot and Typhidot-M tests in febrile Malaysian children. Acta tropica 72: 175–183. 10206117 10.1016/s0001-706x(98)00095-3

[29] Dutta S, Sur D, Manna B, Sen B, Deb AK, et al (2006) Evaluation of new-generation serologic tests for the diagnosis of typhoid fever: data from a community-based surveillance in Calcutta, India. Diagnostic microbiology and infectious disease 56: 359–365. 16938421 10.1016/j.diagmicrobio.2006.06.024

[30] Prakash P, Sen MR, Mishra OP, Gulati AK, Shukla BN, et al (2007) Dot enzyme immunoassay (Typhidot) in diagnosis of typhoid fever in children. Journal of tropical pediatrics 53: 216–217. 17387102 10.1093/tropej/fmm008

[31] Dong B, Galindo CM, Shin E, Acosta CJ, Page AL, et al (2007) Optimizing typhoid fever case definitions by combining serological tests in a large population study in Hechi City, China. Epidemiology and infection 135: 1014–1020. 17217551 10.1017/S0950268806007801 PMC2870657

[32] Begum Z, Hossain MA, Musa AK, Shamsuzzaman AK, Mahmud MC, et al (2009) Comparison between DOT EIA IgM and Widal Test as early diagnosis of typhoid fever. Mymensingh medical journal : MMJ 18: 13–17. 19182742

[33] Beig FK, Ahmad F, Ekram M, Shukla I (2010) Typhidot M and Diazo test vis-a-vis blood culture and Widal test in the early diagnosis of typhoid fever in children in a resource poor setting. The Brazilian journal of infectious diseases : an official publication of the Brazilian Society of Infectious Diseases 14: 589–593. 10.1016/s1413-8670(10)70116-1 21340299

[34] Narayanappa D, Sripathi R, Jagdishkumar K, Rajani HS (2010) Comparative study of dot enzyme immunoassay (Typhidot-M) and Widal test in the diagnosis of typhoid fever. Indian pediatrics 47: 331–333. 19430063 10.1007/s13312-010-0062-x

[35] Fadeel MA, House BL, Wasfy MM, Klena JD, Habashy EE, et al (2011) Evaluation of a newly developed ELISA against Widal, TUBEX-TF and Typhidot for typhoid fever surveillance. Journal of infection in developing countries 5: 169–175. 21444985 10.3855/jidc.1339

[36] Ley B, Thriemer K, Ame SM, Mtove GM, von Seidlein L, et al (2011) Assessment and comparative analysis of a rapid diagnostic test (Tubex(R)) for the diagnosis of typhoid fever among hospitalized children in rural Tanzania. BMC infectious diseases 11: 147. 21609455 10.1186/1471-2334-11-147 PMC3123569

[37] Keddy KH, Sooka A, Letsoalo ME, Hoyland G, Chaignat CL, et al (2011) Sensitivity and specificity of typhoid fever rapid antibody tests for laboratory diagnosis at two sub-Saharan African sites. Bulletin of the World Health Organization 89: 640–647. 21897484 10.2471/BLT.11.087627 PMC3165980

[38] Jesudason M, Esther E, Mathai E (2002) Typhidot test to detect IgG & IgM antibodies in typhoid fever. The Indian journal of medical research 116: 70–72. 12592993

[39] Jesudason MV, Sivakumar S (2006) Prospective evaluation of a rapid diagnostic test Typhidot for typhoid fever. The Indian journal of medical research 123: 513–516. 16783041

[40] WHO (2012) WHO Global Malaria Programme. Information nore on recommended selection criteria for procurement of malaria rapid diagnostic tests (RDTs).

[41] Parry CM, Wijedoru L, Arjyal A, Baker S (2011) The utility of diagnostic tests for enteric fever in endemic locations. Expert review of anti-infective therapy 9: 711–725. 21692675 10.1586/eri.11.47

[42] Program WGM (2011) Good practices for selecting and procuring rapid diagnositc tests for malaria.

[43] WHO (2011) Overview of the prequalification of diagnostic assessment process.

